# Human amniotic fluid stem cells as a model for functional studies of genes involved in human genetic diseases or oncogenesis

**DOI:** 10.18632/oncotarget.328

**Published:** 2011-09-14

**Authors:** Margit Rosner, Helmut Dolznig, Katharina Schipany, Mario Mikula, Oliver Brandau, Markus Hengstschläger

**Affiliations:** ^1^ Medical Genetics, Medical University of Vienna, Währinger Strasse 10, 1090 Vienna, Austria

**Keywords:** stem cells, human genetics, oncogenesis, amniocentesis, RNA interference, mTOR

## Abstract

Besides their putative usage for therapies, stem cells are a promising tool for functional studies of genes involved in human genetic diseases or oncogenesis. For this purpose induced pluripotent stem (iPS) cells can be derived from patients harbouring specific mutations. In contrast to adult stem cells, iPS cells are pluripotent and can efficiently be grown in culture. However, iPS cells are modulated due to the ectopic induction of pluripotency, harbour other somatic mutations accumulated during the life span of the source cells, exhibit only imperfectly cleared epigenetic memory of the source cell, and are often genomically instable. In addition, iPS cells from patients only allow the investigation of mutations, which are not prenatally lethal. Embryonic stem (ES) cells have a high proliferation and differentiation potential, but raise ethical issues. Human embryos, which are not transferred in the course of *in vitro* fertilization, because of preimplantation genetic diagnosis of a genetic defect, are still rarely donated for the establishment of ES cell lines. In addition, their usage for studies on gene functions for oncogenesis is hampered by the fact the ES cells are already tumorigenic *per se*. In 2003 amniotic fluid stem (AFS) cells have been discovered, which meanwhile have been demonstrated to harbour the potential to differentiate into cells of all three germ layers. Monoclonal human AFS cell lines derived from amniocenteses have a high proliferative potential, are genomically stable and are not associated with ethical controversies. Worldwide amniocenteses are performed for routine human genetic diagnosis. We here discuss how generation and banking of monoclonal human AFS cell lines with specific chromosomal aberrations or monogenic disease mutations would allow to study the functional consequences of disease causing mutations. In addition, recently a protocol for efficient and highly reproducible siRNA-mediated long-term knockdown of endogenous gene functions in AFS cells was established. Since AFS cells are not tumorigenic, gene modulations not only allow to investigate the role of endogenous genes involved in human genetic diseases but also may help to reveal putative oncogenic gene functions in different biological models, both *in vitro* and *in vivo*. This concept is discussed and a “proof of principle”, already obtained via modulating genes involved in the mammalian target of rapamycin (mTOR) pathway in AFS cells, is presented.

## INTRODUCTION

In medical genetics the future development of new prophylactic and therapeutic strategies directly depends on a better understanding of the mechanisms by which genetic variation contributes to disease. It must be a major goal of human genetics to use optimal biological models, that allow the investigation of the consequences of a specific genetic aberration. On the one hand, in the past a powerful approach was the establishment of gain- or loss-of-function mouse mutants. However, due to fundamental biological differences many phenotypes of human genetic diseases fail to be successfully replicated in mice. On the other hand, human clinical studies can *per se* also only provide limited information: 1) It is almost impossible to obtain the specific tissue of interest (e.g. neuronal or cardiac cells), given it is not blood. 2) For more detailed biochemical and cell biological investigations a distinct quantity of material must be available. Unfortunately, long expansion of human biopsy material is often accompanied by the induction of mutations in cell culture. Accordingly, today, human stem cells are in the focus of interest of medical genetics [1, 2].

To be useful in medical genetics human stem cells must fulfil certain criteria. They should be available with specific natural occurring genetic aberrations, which are of relevance for certain human pathological phenotypes. In addition, they should harbour high proliferative activities and pluripotency, the potential to differentiate in cells of all three germ layers. Human ES cells are derived from the inner cell mass of the blastocyst. They can be grown in culture in an undifferentiated state and retain their pluripotent differentiation capacity for an unlimited period of time. Since over twenty years human embryos are diagnosed via preimplantation genetic diagnosis in the course of *in vitro* fertilization. This is offered to couples, whose potential offsprings are at risk of single gene disorders or structural and numerical chromosome aberrations [3]. As a consequence, embryos with all kinds of numerical chromosomal abnormalities and unbalanced chromosomal translocations, as well as with specific monogenic disease mutations are excluded from transfer into the uterus and could be used to generate human ES cell lines. The top ten of monogenic diseases diagnosed has been relatively constant over the years, including cystic fibrosis, β-thalassaemia, spinal muscular atrophy, sickle cell disease, Huntington's disease, myotonic dystrophy, Charcot-Marie-Tooth disease, fragile X syndrome, Duchenne muscular dystrophy and haemophilia [3, 4].

In countries, where it is legal to destroy human embryos for research, ES cell lines carrying certain inherited defects have been generated [see e.g. 5, 6]. The fact, that ES cell lines harbouring natural occurring mutations have a great potential in the research on the pathophysiology of these diseases, as well as for the development of new therapies [7], has initiated the creation of different registries for human ES cells [8-10; see also http://www.hescreg.eu]. However, ES cells have certain disadvantages. Firstly, destroying a human embryo for research raises ethical issues, regarding when human life begins and the moral status of a few-days-old embryos. In addition, the spectrum of ES cells carrying an inherited defect is limited due to the fact that *in vitro* fertilization with preimplantation genetic diagnosis is only rarely applied [11-14]. And last but not least, ES cells cannot be used to investigate the role of specific mutations in tumorigenesis in *in vivo* models, since when ES cells are transplanted in mice, they are already *per se* tumorigenic [15].

A second stem cell type, which fulfils the criterias described above, are iPS cells. In 2006, Yamanaka's group demonstrated for the first time that adult mouse fibroblasts can be reprogrammed to pluripotent stem cells via ectopic overexpression of the genes *Pou5f1*, *Sox2*, *Klf4* and *cMyc*, which are known to be expressed in ES cells [16]. Such iPS cells can be grown in culture maintaining their pluripotency to form all three embryonic tissue types. In 2008, the generation of iPS cells from patients with specific genetic diseases was first described [17,18]. Inbetween, a variety of iPS lines from single-gene disorders, chromosome syndromes and complex diseases, including e.g. amyotrophic lateral sclerosis, diabetes mellitus, Parkinson's diseases, Down syndrome, epidermolysis bullosa, or Rett syndrome, have been generated [1, 2, 17-20]. Still, iPS cells also harbour relevant disadvantages. Using cells from adult patients has the advantage that the detailed clinical history of the patient is known, but one must take into account that such cells might have already accumulated other mutations without relevance for the disease of interest. And clearly, such an approach only allows to study mutations, which are not prenatally lethal. Importantly, the process of ectopic induction of pluripotency might also negatively affect the usefulness of these cells as models for human diseases [1, 2, 19, 20]. In addition, recently it was demonstrated that the epigenetic memory of the original differentiated state is not perfectly erased during reprogramming [21, 22]. And finally, it seems to be inevitable that iPS cells accumulate karyotypic abnormalities and gene mutations during propagation in culture [23-26]. We here discuss that human AFS cells might be a powerful alternative for disease modelling.

## HUMAN AFS CELLS TO STUDY NATURAL OCCURRING DISEASE CAUSING MUTATIONS

**Figure 1:**
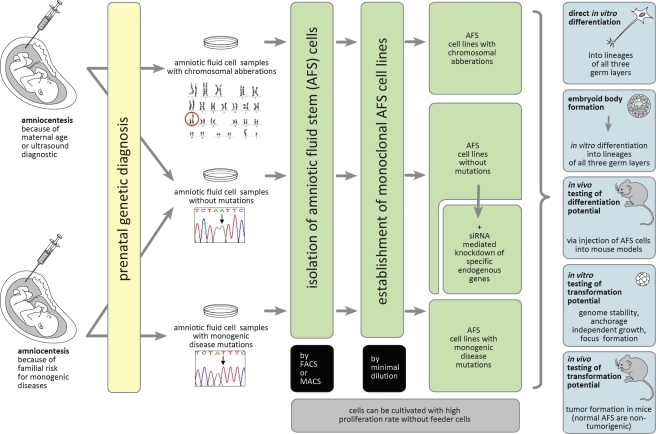
Human AFS cells as a biological model to study gene functions Monoclonal human AFS cell lines can be established from amniocentesis performed because of different medical indications. These stem cell lines can be used for *in vitro* and *in vivo* investigations of the consequences of the functional loss of specific endogenous genes for differentiation and oncogenic processes. For details see the text.

Ultrasound investigations and maternal serum screening are routinely used in prenatal diagnosis. Specific ultrasound signs and/or maternal age are classical indications for invasive genetic prenatal diagnosis, mostly to detect fetal numerical chromosomal aberrations. Invasive prenatal diagnosis is also recommended to detect unbalanced segregation of e.g. balanced parental chromosome translocations. In addition, prenatal chromosome testing also leads to the detection of a major category of “unexpected” chromosomal abnormalities, including numerical aneuploidies other than trisomies 13, 18, and 21, and the wide variety of possible *de novo* structural chromosomal aberrations. Since the Human Genome Project was officially launched the knowledge on single-gene disorders has grown dramatically. Today, a wide variety of mutations, causatively involved in monogenic diseases, can be prenatally diagnosed. Predominantly such prenatal diagnoses are performed as a consequence of a specific family history (Figure 1) [27-29]. Besides other invasive approaches, amniocentesis is a widespreadly accepted standard procedure of care since the 1970s. It is almost unpredictable how many amniocenteses are worldwide performed per year. An older study reported that more than 200.000 amniocentesis procedures were performed already 20 years ago, only in one year (1990) and only in the United States [30, 31].

Despite this wide and well established usage of human amniotic fluid cells in routine prenatal diagnosis, the knowledge about the cells contained in amniotic fluid remained rather elusive [32, 33]. New interest in these cells was initiated in 2001, when Fauza's group reported that amniotic fluid cells could be used in tissue engineering approaches for the surgical repair of congenital anomalies in the perinatal period [34]. This was also about the time when the first suggestion of human amniotic fluid as a new putative source for stem cells was reported [35]. In 2003, a highly proliferative cell type, expressing the pluripotent stem cell marker Oct4, was discovered to exist in human amniotic fluid, providing the first evidence for AFS cells [36]. The confirmation of Oct4-positive cells in amniotic fluid by different other groups then initiated a fast growing stem cell research field [37-45]. In the last years, it became evident that AFS cells can differentiate into cells of all three embryonic tissue types. AFS cell differentiation e.g. upon hematopoietic, neurogenic, osteogenic, chondrogenic, adipogenic, renal and hepatic lineages has been demonstrated [37-63]. Descending from one single Oct4- and NANOG-positive AFS cell, it was possible to induce adipogenic, osteogenic and neurogenic differentiation [47]. In another study, isolation of monoclonal AFS cells, which expressed the markers Oct4 and CD117 (c-kit), allowed to demonstrate that adipogenic, osteogenic, myogenic, endothelial, neurogenic and hepatic cell differentiation could be induced [42]. Monoclonal human AFS cells can form embryoid bodies (EBs) when cultured without anti-differentiation factors under conditions in which they are unable to attach to the surface of culture dishes and without contact to feeder cells. The formation of such three-dimensional multicellular aggregates is accompanied by a decrease of stem cell marker expression and by the induction of differentiation into different lineages [64].

Taken together, these findings prove that human AFS cells are pluripotent and able to form embryoid bodies and to differentiate into cell types of all three germ layers. These data were obtained using monoclonal human AFS cell lines generated via magnetic cell sorting and minimal dilution approaches from amniocentesis samples. So established monoclonal lines can be expanded as immature stem cells with high proliferation rate in culture without the need of feeder cells [42, 60, 61, 64]. We here suggest the generation and banking of normal human AFS cell lines, of AFS cell lines with chromosomal aberrations, as well as of AFS cell lines with specific monogenic disease mutations for research purposes (Figure 1). Such lines carrying natural occurring mutations could provide an optimal tool for disease modelling with the aim to obtain more detailed insights into the functions of the involved chromosomal regions or genes. As in the past already performed with normal monoclonal human AFS cell lines [42, 58, 60, 61, 63, 64], lines harbouring mutations could be investigated in *in vitro* differentiation experiments (including e.g. organotypic reaggregation assays, see below), in embryoid body formation assays, and could be investigated in mouse models *in vivo* (Figure 1). Adult stem cells exhibit lower differentiation potential than AFS cells and cannot be grown with high proliferative activity. Compared to ES cells, AFS cells do not raise ethical issues and the spectrum of samples with pathogenic mutations should be larger. Compared to iPS cells, there is no need for ectopic induction of pluripotency in AFS cells. They are genomically stable, they do neither harbour the epigenetic memory nor somatic mutations of already differentiated source cells, and mutations, which allow implantation but are not compatible with a prenatal development until birth, could also be included [1, 12, 19, 20, 42-45, 55-57, 60, 63-65]. Accordingly, AFS cells could provide an optimal tool for basic research and disease modelling in medical genetics (Figure 1).

## HUMAN AFS CELLS TO INVESTIGATE GENES INVOLVED IN ONCOGENESIS

Stem cells could also be a powerful tool to study the consequences of genetic modulations for transformation processes, both, *in vitro* e.g. in colony formation assays and *in vivo* in mouse models (Figure 1). Such approaches are hampered when the chosen stem cell type, in its undifferentiated form, is tumorigenic *per se*. This holds true for ES cells [1, 13-15]. Similarly, in the study describing the first generation of iPS cells the authors reported that subcutaneous transplantation of these stem cells into nude mice also resulted in tumor development [16]. Recently, it became clear that the tumor-forming propensities of the many different types of iPS cells vary significantly. This is due to the different methods used for reprogramming (the genes or proteins, which have been modulated), the different iPS cells' tissues of origin, the different genetic background of the donor (including the somatic mutations accumulated during the life span of the source cell), and very likely also to the propensity of iPS cells to accumulate mutations during cultivation [1, 19, 66, 67]. Accordingly, although the question whether ES or iPS cells, after differentiation along specific lineages, could be used for studies on transformation processes still needs to be further investigated, it is obvious that such experimental approaches would have to face many difficulties and limitations, which might negatively affect the level of interpretation.

AFS cells are primary cells of a very early stage of human development. Accordingly, they very likely did not have time to accumulate many somatic mutations yet [44, 45, 55-57]. In addition, the investigations performed so far revealed that monoclonal human AFS cell lines maintain genome stability during expansion [42, 64]. And most importantly, AFS cells, unlike ES and iPS cells, do not induce tumor formation in severe combined immunodeficient mice [42]. Accordingly, we here want to suggest the usage of different AFS cell lines to investigate the role of specific genetic modulations for transformation processes and tumor development (Figure 1). Although possible, we would not favour the ectopic expression of oncogenes in AFS cells, because of the risk that the so obtained high ectopic levels of expression would not necessarily mimick a natural occurring situation. We rather want to suggest the usage of siRNA-mediated knockdown of endogenous tumor suppressor genes (for examples see below). Monoclonal human AFS cells with so downregulated endogenous gene functions could then be investigated in *in vitro* and *in vivo* transformation assays as indicated in Figure 1. For this purpose we have recently established a protocol for efficient siRNA-mediated prolonged gene silencing in AFS cells. This protocol allows a 96-98% downregulation of the endogenous expression of tumor suppressors, such as the TSC2 gene product tuberin or the cyclin-dependent kinase inhibitor p27, over a time period of about 14 days [68].

## MODULATION OF MTOR CASCADE COMPONENTS IN HUMAN AFS CELLS

Very recently we already made use of the approach discussed above to functionally investigate the role of components of the mTOR signalling cascade in human AFS cells. mTOR is the key component of the insulin signalling cascade, which is involved in a wide variety of different processes such as cell growth, proliferation, metabolism, transcription, translation, survival, autophagy, aging, differentiation and oncogenesis. An important upstream regulator of mTOR is the oncogenic kinase Akt, which itself is activated via the enzyme cascade of PI3K (phosphatidylinositol-3-kinase) and PDK1 (phosphoinositide-dependent kinase-1). Akt-triggered phosphorylation of the tumor suppressor protein tuberin (TSC2) downregulates its GTPase-activating potential toward Rheb, which is a potent regulator of mTOR. In mammalian cells, two mTOR containing complexes are described, mTORC1 (consisting of mTOR, raptor and mLST8) and mTORC2 (containing mTOR, mLST8, rictor and sin1). mTORC1 is involved in the control of mRNA translation e.g. via phosphorylating the p70S6Kinase, whereas mTORC2 phosphorylates and activates Akt [69-72]. Deregulation of upstream regulators of mTOR, such as e.g. Wnt, Ras, TNF-α, PI3K, or Akt is a hallmark in many human cancers. Mutations in the mTOR pathway component genes *TSC1, TSC2, LKB1, PTEN, VHL, NF1* and *PKD1* trigger the development of the human genetic syndromes Tuberous sclerosis, Peutz-Jeghers syndrome, Cowden syndrome, Bannayan-Riley-Ruvalcaba syndrome, Lhermitte-Duclos disease, Proteus syndrome, von Hippel-Lindau disease, Neurofibromatosis type 1, and Polycystic kidney disease. Besides a variety of single gene disorders and tumorigenesis, the mTOR pathway has also been shown to be of relevance for the development of complex diseases, such as cardiac hypertrophy, obesity or type 2 diabetes [73-75].

Studies performed in hematopoietic stem cells provided first evidence that mTOR might also play a role in stem cell physiology [76]. A major characteristic of pluripotent stem cells is their potential to spontaneously form EBs. EB formation is a commonly used *in vitro* approach to recapitulate and investigate the three-dimensional and tissue level contexts of the cell differentiation phenomena mimicking early mammalian embryogenesis [77, 78]. Since it was already earlier shown that the mTOR cascade is fully active in human AFS cells [79], we induced EB formation of monoclonal human AFS cell lines to investigate the role of mTOR. siRNA-mediated knockdown of raptor or rictor demonstrated that EB formation depends on both, mTORC1 and mTORC2 [64]. Together with similar results in human ES cells [80], these findings were the first demonstration of the role of mTOR for EB formation of pluripotent stem cells.

We have further shown that human monoclonal AFS cells are able to contribute to the formation process of renal tissues. For this purpose, murine embryonic kidneys were dissociated into a single-cell suspension and then reaggregated to form organotypic renal structures. Using this approach it was possible to form chimeric renal structures via mixing murine embryonic kidney cells with monoclonal human AFS cells selected from amniocentesis samples. Monoclonal, pluripotent Oct4- and CD117-positive AFS cells were able to participate in the formation process of different renal tissues accompanied by the induction of the expression of well known renal markers [60]. In this study the highly efficient and reproducible protocol for prolonged siRNA-mediated gene silencing in human monoclonal AFS cells [68], which was used for the EB study described above, also allowed to demonstrate the pivotal role of mTOR component genes for renal tissue formation [60].

Taken together the approach of siRNA-mediated knockdown of mTOR cascade components in monoclonal human AFS cell lines allowed to study the consequences on EB formation and on the potential to differentiate along renal lineages. These investigations already provided a “proof of principle” for the here discussed usage of human AFS cells as a model for functional studies of specific genes.
